# Precision Medicine in Graves’ Disease and Ophthalmopathy

**DOI:** 10.3389/fphar.2021.754386

**Published:** 2021-10-28

**Authors:** Giusy Elia, Poupak Fallahi, Francesca Ragusa, Sabrina Rosaria Paparo, Valeria Mazzi, Salvatore Benvenga, Alessandro Antonelli, Silvia Martina Ferrari

**Affiliations:** ^1^ Department of Surgical, Medical and Molecular Pathology and Critical Area, University of Pisa, Pisa, Italy; ^2^ Department of Translational Research and New Technologies in Medicine and Surgery, University of Pisa, Pisa, Italy; ^3^ Department of Clinical and Experimental Medicine, University of Pisa, Pisa, Italy; ^4^ Department of Clinical and Experimental Medicine, University of Messina, Messina, Italy; ^5^ Master Program on Childhood, Adolescent and Women’s Endocrine Health, University of Messina, Messina, Italy; ^6^ Interdepartmental Program of Molecular and Clinical Endocrinology and Women’s Endocrine Health, University Hospital, A.O.U. Policlinico G. Martino, Messina, Italy

**Keywords:** Graves’ disease, Graves’ ophthalmology, thyroid eye disease, teprotumumab, tocilizumab, rituximab, chemokine, cytokines

## Abstract

Graves’ disease (GD) is a condition caused by an autoimmune process involving the thyroid gland, whose main outcome is hyperthyroidism. TSAb start the autoimmune process stimulating the overproduction of thyroid hormones. In addition, TSAb can stimulate TSH-R expressed in fibroblasts and orbital pre-adipocytes leading to the manifestation of Graves’ ophtalmopathy (GO). Also, autoantibodies directed against IGF-1R have an important role in immune-pathogenesis of GO. Fundamental is the role played by cytokines (IFN-γ, TNF-α, Il-6), and Th1 chemokines in the immune-pathogenesis of both disorders, particularly in the active phase. Novel discoveries in the field led to the investigation of promising therapies, such as immune-therapies towards specific antigens (for example against TSH-R), aiming in restoring the immune tolerance versus the immune dominant epitopes associated with autoimmunity in GD. Moreover, Etanercept (that blocks the TNF-mediated inflammatory responses), TCZ (that acts against the IL-6 receptor), and RTX (that acts against CD20) have proven to be useful and safe therapeutic options in refractory GO treatment. Furthermore, teprotumumab (a human monoclonal anti-IGF-1R blocking antibody), have been revealed effective in the treatment of patients with moderate-severe GO and it is now approved for GO therapy in United States. Molecules able to act as antagonists of CXCR3, or to block CXCL10, are also under study. More extensive researches are needed to deepen out these drugs as well as to identify new targeted and effective therapies, that will permit a more precise identification of GD, or GO, patients able to respond to specific targeted therapies.

## Introduction

The immune system has the important role to protect our body from foreign or inner attacks, but unfortunately this delicate mechanism can be broken and our immune system can attack the self-antigens leading to the appearance of autoimmune disorders. Several factors can contribute to this breakdown, such as environmental, genetics, immunological, hormonal conditions, being part of the “mosaic of autoimmunity” (Shoenfeld et al., 2019). Nowadays autoimmune disorders are largely widespread and in rising growth with women representing the mostly affected gender; moreover, autoimmune disorders might run together in the same person (Cooper and Stroehla, 2003; Lerner et al., 2015; Fallahi et al., 2019). The most frequent autoimmune disorders are the autoimmune thyroid disorders (AITD), directed against the thyroid gland, whose main clinical features are Graves' disease (GD), and Hashimoto’s thyroiditis (HT) (Antonelli et al., 2015). Here we review the new pharmacological progresses made for the treatment of GD, and of Graves’ ophtalmopathy (GO).

### Grave’s Disease and Ophtalmopathy

Graves’ disease has a prevalence of about 1–1.5%, in iodine sufficient West countries, with an incidence of 20–30 new cases/100,000 for year. The risk is higher for women, aged 35–55 years, and among African Americans (Smith and Hegedüs, 2016; Kahaly, 2020).

Several factors can predispose to the onset of GD, ranging from genetic, environmental, hormonal conditions to habits such as the smoke. Studies involving twins reinforced the role covered by genetic. A cohort study of 110,814 twins examined co-aggregation and heritability of HT and GD. They observed a higher co-aggregation in monozygotic twins with respect to dizygotic twins, and found a high heritability for GD (Skov et al., 2020). Some variants of the *DRB1*, *DQA1* and *DQB1* genes of the human leukocyte antigen (HLA) class II genes are predictors of the development of GD, whereas others have a protective role, HLA-DRB1* 07, *HLA-C03*, and HLA-C*16 (Vejrazkova et al., 2018; Wémeau et al., 2018). Other immune-competent genes whose variants may be involved in GD are *PTPN22*, *CTLA4*, *CD40*, *FOXP3*, *ARID5*, *NRXN3*, *IKZF3*. Also, other specific thyroid antigens such as “thyroid-stimulating-hormone receptor” (TSH-R), or *Thyroglobulin* (Tg), have been identified by a whole-genome linkage study as major AITD risk genes (Tomer et al., 2003; Vejrazkova et al., 2018). Susceptible individuals could be more easily influenced by environmental triggers, such as external radiation, iodine, selenium, smoking or viruses (Ferrari et al., 2017). Lately, 5 cases of GD reappearance, and a case of GO, after SARS COV-2 infection has been observed (Mateu-Salat et al., 2020; Harris and Al Mushref, 2021; Jiménez-Blanco et al., 2021; Lanzolla et al., 2021).

Therefore, these conditions predispose to the break of the immune tolerance towards thyroid antigens, mainly against the TSH-R. Anti-TSH-R autoantibodies (TRAb) are implicated in the thyroidal and extra-thyroidal manifestations of GD. TRAb are released by B lymphocytes, that infiltrate the thyroid gland during the autoimmune process. They are functionally divided in stimulating (TSAb), blocking (TBAb) and neutral antibodies, with the stimulating ones that induce the hyper-production of thyroid hormones, therefore leading to the clinical manifestations of hyperthyroidism (Smith and Hegedüs, 2016; Antonelli et al., 2020). TSAb have a significant role not only in the thyroid gland, but also in the extra-thyroidal manifestations of GD, such as GO, or pretibial myxedema. Other thyroid antigens are involved in the autoimmune process of GD, such as thyroid peroxydase (TPO) and/or Tg, whose antibodies are found in about 50–70% of cases of GD (Wémeau et al., 2018). The involvement of autoantibodies binding the insulin-like growth factor-1 receptor (IGF-1R) has been found to be implicated in the development of GO (Smith, 2019). They are able to induce the expression of the chemokine “regulated on activation normal T cell expressed and secreted” (RANTES) and IL-16 (Pritchard et al., 2002; Pritchard et al., 2003; Smith, 2019) attracting T lymphocytes, that enter into the site of tissue damage inducing and perpetuating the inflammatory process (Cruikshank et al., 1987; Schall et al., 1988; Smith, 2019).

### Cytokines/Chemokines in GD

Fundamental is also the role covered by “Th1 chemokines” (CXCL10, CXCL9, CXCL11), and their (C-X-C)R3 receptor in the immune-pathogenesis of both disorders. In the active phase of GD prevails a Th1 immune response, in which, subsequently to a CXCL10 production by resident follicular epithelial cells, occurs a recruitment of Th1 cells. This process leads to the initiation, and amplification of the inflammation (Romagnani et al., 2002; Antonelli et al., 2020) **(** Table 1 **).**

**TABLE 1:** Cytokines and/or Chemokines in Graves’ disease and in Graves’ Ophtalmopathy.

Cytokines and/or chemokines	Studies [ref]
IL-6	-sIL-6R concentrations were higher in GD patients with active inflammatory thyroid-associated ophthalmopathy than those in patients with inactive or absent thyroid-associated ophthalmopathy Salvi et al. (1996)
TNF-α/IFN-γ	-IFN-γ, or IFN-γ+TNF-α combination stimulate Th1 chemokines in TFCs Antonelli et al. (2006a); Antonelli et al. (2009); Antonelli et al. (2010); Ferrari et al. (2015); Fallahi et al. (2020)
	-IFN-γ, or IFN-γ+TNF-α combination stimulate Th1 chemokines in the primary cell cultures of retro-bulbar cells of GO patients Antonelli et al. (2006a); Dong et al. (2011)
CXCL10/CXCL9	-Maximal expression of CXCL10 and CXCL9 was found in the thyroid gland of patients with recent-onset GD and was correlated with IFN-γ. High levels of CXCL10 could be measured in the serum of patients with short-duration GD Romagnani et al. (2002)
	-Significant reductions in CXCL9 and CXCL10 serum concentrations during CS and TR treatment as compared both to control group and to basal values in GO patients Mysliwiec et al. (2012)
CXCL10	-Thyrocytes and retrobulbar cell types participate in the self-perpetuation of inflammation by releasing chemokines under the influence of cytokines. PPAR-γ activation plays an inhibitory role in this process Antonelli et al. (2006a)
	-sCXCL10 are associated with the active phase of GD in both newly diagnosed and relapsing hyperthyroid patients. The reduction of sCXCL10 in treated patients with GD may be related to the immunomodulatory effects of MMI Antonelli et al, (2006b)
	- sCXCL10 are higher in newly diagnosed hyperthyroid patients with GD than in those with TNG, and decrease when euthyroidism is achieved with antithyroid therapy Antonelli et al. (2006c)
	- High sCXCL10 are associated with the hyperthyroid phase in GD but not TNG Antonelli et al. (2007)
	- Data show a relationship between serum CXCL10 and GD activity Leite et al. (2011)
	-CXCL10 participates in the early inflammatory response after radioactive iodine therapy in patients with GD and shows a strong association with the autoimmune process Dong et al., 2011
CXCL9/CXCL11	-Thyrocytes and retrobulbar cell types from patients with GD and GO released CXCL9 and CXCL11 chemokines when stimulated with cytokines. PPAR-γ activation plays an inhibitory role in this process Antonelli et al. (2009)
	- PPAR-α has been found in GD and control thyrocytes. PPAR-α activators are potent inhibitors of the secretion of CXCL9 and CXCL11 Antonelli et al. (2010)
	-Serum CXCL9 and CXCL11 levels are associated with the active phase of GD both in newly diagnosed and relapsing hyperthyroid patients. The reduction of serum CXCL9 and CXCL11 levels in GD patients in treatment with MMI, may be related to the immunomodulatory effects of MMI Antonelli et al. (2013)
	-PPAR-α activators inhibit CXCL9 and CXCL11 chemokines in normal and GO fibroblasts and preadipocytes Antonelli et al. (2012).
CXCL10/CCL2	-CCL2 is modulated by IFN-γ and TNF-α in GD and normal thyrocytes. PPAR-α activators inhibit the secretion of CXCL10 and CCL2 in thyrocytes Antonelli et al. (2011)
	-EOM participates in the self-perpetuation of inflammation by releasing CXCL10 and CCL2 chemokines under the influence of cytokines, in GO. PPAR-γ agonist activation plays an inhibitory role on CXCL10, but stimulates the release of CCL2 Antonelli et al. (2014)

CS, Corticosteroids; EOM, extra-ocular muscle; GD, Graves’ disease; GO, Graves’ Ophtalmopathy; IFN, Interferon; IL, interleukin; MMI, methimazole; PPAR, peroxisome proliferator-activated receptor; sCXCL10, Serum levels of CXCL10; TFCs, thyroid follicular cells; TNG, toxic nodular goitre; TR, teleradiotherapy.

In basal condition thyroid follicular cells do not secrete Th1 chemokines, while a release occurs under interferon (IFN)-γ, and it is higher under a combined IFN-γ and tumor necrosis factor (TNF)-α (IFN-γ+TNF-α) stimulation (Antonelli et al., 2006a; Antonelli et al., 2009; Antonelli et al., 2010; Ferrari et al., 2015; Fallahi et al., 2020). Therefore, the cytokines stimulation made thyrocytes largely involved in the inflammatory process through the release of Th1 chemokine. The peroxisome proliferator-activated receptor (PPAR)-γ agonists, such as PPAR-α agonists, instead inhibit this process (Antonelli et al., 2009; Antonelli et al., 2010; Antonelli et al., 2011; Ferrari et al., 2015).

Both the active and the relapse phase of GD is characterized by high circulating Th1 chemokines, that decline with methimazole (MMI) therapy. The immune-modulatory effect of MMI is associated with the decrease of serum CXCL10, that achieves normal levels with thyroid hormones normalization, or with GD in remission. The reduction of circulating CXCL10 was not associated with the reduction of AbTg or AbTPO levels, but with the decrease of TRAb (Antonelli et al., 2006a; Antonelli et al., 2006b; Antonelli et al., 2013).

CXCL10 serum levels were also assessed in GD patients who underwent total thyroidectomy or radioactive iodine (RAI) treatment*.* The decrease of CXCL10 levels following these treatments suggests that the site of production of this chemokine is the thyroid gland itself (Antonelli et al., 2006c; Antonelli et al., 2007; Leite et al., 2011).

Furthermore, a study investigated CXCL10 levels in subjects with: 1) 16 new diagnoses of GD in therapy with MMI; 2) 15 relapsed GD in treatment with RAI; 3) 18 controls. Subjects treated with MMI reported a decline of CXCL10 and euthyroidism after 6 and 12 months; those treated with RAI showed a reduction of CXCL10 levels after 3, 6, 9, and 12 months, with a similar TRAb decrease (Leite et al., 2011).

### Cytokines/Chemokines in GO

The onset of GO and GD are often concomitant, with GO involving almost 30–50% of GD patients. Subjects who are more prone to develop a GO are smoker, or patients with a severe hyperthyroidism, and those with very high levels of TSAb. Athough a primary prevention of GO is not available, the progression from a subclinical condition into overt and/or severe ones can be avoided through an early diagnosis, an accurate control of thyroid function, stop of smoking, and with the early therapy of mild GO (Wiersinga and Bartalena, 2002; Perricone et al., 2016; Smith et al., 2017) (Table 1).

GO retro-bulbar cells (fibroblasts and preadipocytes) are highly involved in the perpetuation of the orbital inflammation by releasing Th1 chemokines (CXCL10, CXL11, CXCL9) under the influence of IFN-γ. Higher serum CXCL10 levels have been observed in GO patients with active disease in comparison to the inactive ones. Moreover, Th1 chemokines were basally absent in the primary cell cultures of retro-bulbar cells of GO patients; whereas their release was stimulated by IFN-γ, or IFN-γ+TNF-α stimulation (Antonelli et al., 2006a; Dong et al., 2011). PPAR-α, -δ, and -γ are found in GO fibroblasts or preadipocytes, and the PPAR-γ agonists showed an inhibitory role on Th1 chemokines release (Antonelli et al., 2009; Antonelli et al., 2012).

Another study explored the involvement of retro-bulbar myoblasts in the immune-pathogenesis of GO (Antonelli et al., 2014). High serum CXCL10 levels have been observed in both patients having active GO associated with extraocular muscle (EOM) or with orbital fat involvement, in comparison with controls. CXCL10 was not detectable in primary EOM cells from GO patients, whereas it was released under the cytokines stimulation (IFN-γ and/or TNF-α). Therefore, EOM are involved in the inflammatory GO process through the release of Th1 chemokines (Antonelli et al., 2014).

A potential use of Th1 chemokines as markers of GO activity has been investigated by a study that involved forty-two GO subjects of which: 20 were GD patients (half in euthyroidism and half in hyperthyroidism); 15 GO patients in euthyroidism [previously treated with intravenous of methylprednisolone (ivMP) and teleradiotherapy], and seven were controls. Interestingly, a significant decrease of Th1 chemokines occurred after ivMP and teleradiotherapy. The reduction of circulating Th1 chemokines was not associated with the reduction of AbTg or AbTPO levels, nor of TRAb.

Therefore, these chemokines may aid in the therapeutic decision-making of GO patients (Mysliwiec et al., 2012).

## Therapy for GD

### Antithyroid Drugs

MMI, carbimazole, and propylthiouracil (PTU) are the first-choice therapy for GD. These drugs act by inhibiting TPO, and blocking the synthesis of thyroid hormones. PTU also blocks extrathyroidal deiodination of T4 to T3. The toxicity profile of these drugs makes them the preferred with respect to radioiodine (Bartalena et al., 2016; Burch and Cooper, 2015; Antonelli et al., 2020), however the risk of relapse after therapies is high. Furthermore, MMI and PTU have immune-modulatory effect reducing TSAb levels (Antonelli et al., 2013).

### Radioiodine Therapy

RAI has been widely used; it gives relief from symptoms of hyperthyroidism within weeks. Antithyroid drugs can be suspended 3–7 days before and after radioiodine in order to improve its effectiveness. However, radioiodine can cause or worsen GO. Therefore, a close monitoring of the thyroid function should be performed, and when hypothyroidism occurs, it needs to be treated as soon as possible (Galetta et al., 2008; Smith and Hegedüs, 2016).

### Surgery

Surgery is needed in particular conditions, such as if the patient do not want to receive anti-thyroid drugs, or radioiodine; in presence of a large goiter; and for women who would like to have pregnancy. The patients must reach euthyroidism, before they can undergo surgery. This will reduce the risk of complications (Feroci et al., 2014; Smith and Hegedüs, 2016).

### Antigen-specific Immunotherapy

The antigen-specific immunotherapies aim to re-establish an immunological tolerance against the immune dominant epitopes involved in autoimmunity, without inducing generalized immunosuppression (Pearce et al., 2019). A study investigated a combination of two TSHR peptides (ATX-GD-59) in 12 subjects with mild-to-moderate untreated hyperthyroidism. A potential efficacy of this treatment has been suggested; 70% of the treated subjects reported an improvement in free thyroid hormones (Pearce et al., 2019).

## Therapy for GO

### Corticosteroids Therapy

The common treatment for active GO are high-dose of ivMP. A multicenter trial demonstrated the effectiveness of ivMP in improving inflammation in about 80–70% of the cases, and eye muscle function in 50%. Nevertheless, about 20% were no significantly responders to the treatment, and progression disease or compression of the optic nerve occurred in about 4% of the subjects (Bartalena et al., 2012).

Therefore, new targets involved in the autoimmune reaction have been taken in accounts for the development of new drugs, such as TSH-R, the IGF-1R (on fibroblasts), T or B lymphocytes, chemokines and cytokines (Fallahi et al., 2016).

### TSH-R Antagonists

Drugs acting against TSH-R have been recently investigated. Promising results have been obtained by a molecule NCGC0022960 able to reduce the production of hyaluronic acid in primary cell culture of retro-orbital fibroblasts/adipocytes of GO (Emerson, 2011; Turcu et al., 2013).

In a patients with follicular thyroid cancer (FTC), GD, with high levels of TSAb, and severe GO, was tested K1-70 a monoclonal antibody anti-TSHR. After the start of the therapy, the TSAb activity decreased and GO improved. Moreover, on K1-70 monotherapy during the pause in lenvatinib, used for the treatment of FTC, occurs a stabilization of several metastatic lesions (Ryder et al., 2021).

### Etanercept and Tocilizumab

Cytokines are largely involved in the autoimmune process of the GO. TNF-α and IL-6 have a crucial role in this process (Bahn, 2010).

Etanercept is a dimeric protein able to bind two molecules of TNF, avoiding its interaction with receptors on the cell surface, and subsequently the TNF-mediated inflammatory responses. This molecule is the choice option for different autoimmune disorders [e.g. rheumatoid arthritis (RA), ankylosing spondylitis in adults, and juvenile idiopathic arthritis or plaque psoriasis in paediatric patients] (Scott, 2014). In a pilot study the efficacy of etanercept was investigated in 10 GO subjects (25 mg twice weekly, were administered for 12 weeks). An improvement was observed in 60% of patients; a reactivation of GO occurred in three patients after cessation. No serious adverse events (AEs) or side effects were registered during a follow-up of 18 months (Paridaens et al., 2005). Another paper reported a case of a patient with RA and GO. She was treated with etanercept for RA achieving also a clinical improvement of GO symptoms (Boskovic et al., 2019). Additional researches are needed in order to evaluate the effectiveness of TNF-α inhibitors, and to compare its side effects with the current medical treatment.

The cytokine IL-6 is released by T lymphocytes and macrophages and has a pro-inflammatory activity. GO patients in the active phase showed increased levels of circulating IL-6 and of its receptor (Salvi et al., 1996). The monoclonal antibody (mAb) tocilizumab (TCZ) acts against the IL-6 receptor, and received the approval for the treatment of RA, systemic juvenile idiopathic arthritis and Castleman’s disease (Emery et al., 2008; Yokota et al., 2008). TCZ was evaluated in 18 GO patients, not responders to corticosteroids (CS). Thirteen patients showed a decreased proptosis, fifteen had an improvement of the extraocular motility and seven out of 13 resolved their diplopia (Pérez-Moreiras et al., 2014). Another open-label multicenter study assessed the effectiveness of TCZ enrolling 48 patients with glucocorticoid-resistant GO (Sánchez-Bilbao et al., 2020). The follow-up lasts for a mean of 16.1 ± 2.1 months, and it was observed a decrease of disease activity [Clinical Activity Score (CAS) ≤ 3] in many patients; TCZ was withdrawn in 29 cases, because of low disease activity in 25 cases, or inefficacy in four subjects. No serious AEs were registered. Thereby, TCZ appears an efficacy, useful and safe therapeutic option in refractory GO treatment (Sánchez-Bilbao et al., 2020).

### Rituximab

Rituximab (RTX) acts against CD20 placed on B cells; thereby it induces B cells death, and is indicated in the therapy of those diseases characterized by elevated levels of B-lymphocytes or dysfunctional B-lymphocytes, and overactive B-cells. This mAb has no effect on plasma cells, it doesn’t interfere with the antibody synthesis (Ahuja et al., 2008). Since RTX reduces the number of B lymphocytes, the burden of cytokines and the secreted autoantibodies, it has been suggested for the treatment of GO (Salvi et al., 2012). Conflicting results were reported about the efficacy of RTX in GO.

A study included 25 GO subjects in a prospective, placebo-controlled, randomized trial (Stan et al., 2015); patients received two RTX infusions, or two saline infusions, 2 weeks apart. RTX appeared not effective in GO, because no differences were registered about the improvement of CAS with respect to placebo (Stan et al., 2015).

However, another double-blind, randomized trial enrolling 32 subjects reported different outcomes. The patients received RTX or ivMP; 100% of RTX patients achieved an improvement at 24 weeks, compared to 69% after ivMP, therefore assessing a higher efficacy of RTX than ivMP in GO patients (Salvi et al., 2015).

Recently a multicenter retrospective study (Deltour et al., 2020) investigated the efficacy of RTX in forty GO patients resistant to CS, or in cases of CS dependence. The Authors found that RTX is effective as a second-line treatment of these patients, especially if the disease is recent and active; and when it is administered in the early phase of the disease. The time of administration may explain the contradictory results obtained in the previous randomized studies (Salvi et al., 2015; Stan et al., 2015; Deltour et al., 2020).

### Teprotumumab

An overexpression of IGF-1R has been found in orbital connective tissues, T and B cells in GD and GO. GD patients generated autoantibodies that are able to bind to IGF-1R and initiate the signaling from the TSHR/IGF-1R physical and functional protein complex. Therefore, the use of mAbs against IGF-1R may attenuate signaling from either TSHR or IGF-1R (Smith, 2021).

Teprotumumab (RV 001, R1507) is a human monoclonal anti-IGF-1R blocking antibody. An *in vitro* study, showed its efficacy in reducing the fibrocyte display of IGF-1R and TSH-R, such as their downstream signals, blocking the induction of pro-inflammatory cytokines (Chen et al., 2014).

A first multicenter, double-masked, randomized, placebo-controlled trial was carried out to investigate the efficacy of teprotumumab in patients with active, moderate-to-severe ophthalmopathy (Smith et al., 2017). The 88 enrolled subjects were randomly assigned to the placebo group or to the teprotumumab group. 69% of patients of the teprotumumab group had a response at week 24 (*p* < 0.001), with respect to the 20% of the placebo group. Moreover, the response was rapidly achieved (*p* < 0.001) in the teprotumumab group, 43% at week 6, against only 4% of the placebo. These findings supported the efficacy of this drug in reducing proptosis and CAS in patients with active GO (Smith et al., 2017).

A subsequent randomized, double-masked, placebo-controlled, phase 3, multicenter trial, involved 83 patients with moderate to severe GO (with a duration of GO < 9 months), of whom 41 received teprotumumab and 42 placebo. Teprotumumab led to better outcomes in proptosis, CAS, diplopia, and quality of life than placebo; serious AEs were uncommon (Douglas et al., 2020). Additionaly, the non responders were included in an extension of the phase 3 trial; they received teprotumumab as an open label, regardless of whether or not they had received the active drug, or placebo, during the 24-weeks treatment phase (Smith, 2021). The response to the therapy occurs in a similar fraction of patients, such as in the initial intervention. Follow-up data (of phase 3 trial, plus extension study) revealed that the majority of patients who responded with amelioration of proptosis and diplopia at week 24 maintained their responses (56 and 58%, respectively) (Smith, 2021).

These studies showed the efficacy of teprotumumab as well as its safety. The AEs encountered were mild to moderate in severity, with the most common being hyperglycemia found especially in patients with diabetes, and easily managed by adjusting the therapy. Other AEs were muscle cramps, hearing abnormalities, hair loss, diarrhea and dysgeusia (Chen et al., 2014; Smith et al., 2017; Smith, 2021).

Teprotumumab has been approved by the US FDA for the therapy of GO, and is now in clinical use in North America (Smith, 2021).

However, more studies are needed to assess its effectiveness in such conditions e.g. patients with a chronic and less active GO condition or with an impaired vision due to compressive optic neuropathy (Markham, 2020; Smith, 2021).

## Conclusion

Graves’ disease (GD) is a condition caused by an autoimmune process involving the thyroid gland, whose main outcome is hyperthyroidism. TSAb start the autoimmune process stimulating the overproduction of thyroid hormones. In addition, TSAb can stimulate TSH-R expressed in fibroblasts and orbital pre-adipocytes, leading to the manifestation of GO. Also, autoantibodies directed against IGF-1R have an important role in immune-pathogenesis of GO. Fundamental is the role played by cytokines (IFN-γ, TNF-α, Il-6), and Th1 chemokines in the immune-pathogenesis of both disorders, particularly in the active phase.

Novel discoveries **(** Table 2 **)** in the field led to the investigation of promising therapies, such as immune-therapies towards specific antigens (for example against TSH-R), aiming in restoring the immune tolerance in GD.

**TABLE 2:** Latest drugs for Graves’ Ophtalmopathy.

Drugs	Molecular targets	Studies [ref]
NCGC0022960	TSH-R	Reduced production of hyaluronic acid in primary cell culture of retro-orbital fibroblasts/adipocytes of GO Turcu et al. (2013)
K1-70	TSH-R	-Case report; TSAb activity decreased and GO improved Ryder et al. (2021)
Etanercept	TNF-α	-Pilot study; improvement in 60% of pts Paridaens et al. (2005)
		-Case report patient with RA and GO: clinical improvement of GO symptoms Boskovic et al (2019)
Tocilizumab	IL-6	- 18 pts, not responders to corticosteroids. Thirteen pts showed a decreased proptosis, fifteen had an improvement of the extraocular motility and 7 out of 13 resolved their diplopia Pérez Moreiras et al., (2014)
		-An open-label multicenter study including 48 pts with glucocorticoid-resistant GO. Decrease of disease activity was registered in many pts; TCZ was withdrawn in 29 pts, because of low disease activity in 25 cases, or inefficacy in 4 subjects. Sánchez-Bilbao et al. (2020)
Rituximab	CD20 on B cells	-25 GO subjects in a prospective, placebo-controlled, randomized trial; RTX appeared not effective in GO, because no differences were registered about the improvement of CAS with respect to placebo Stan et al. (2015)
		- A double-blind, randomized trial enrolling 32 subjects. The pts received RTX or ivMP; 100% of RTX pts achieved an improvement at 24 weeks, compared to 69% after ivMP Salvi et al. (2015)
		-A multicenter retrospective study involving forty GO pts resistant to CS, or having CS dependence. RTX appeared effective as a second-line treatment of these pts, especially if the disease is recent and active; and when it is administered in the early phase of the disease Deltour et al. (2020)
Teprotumumab	IGF-1R	-A first multicenter, double-masked, randomized, placebo-controlled trial involved pts with active, moderate-to-severe ophthalmopathy. The 88 enrolled subjects were randomly assigned to the placebo group or to the teprotumumab group. 69% of pts of the teprotumumab group had a response at week 24 (*p* < 0.001), with respect to the 20% of the placebo group. The response was rapidly achieved (*p* < 0.001) in the teprotumumab group, 43% at week 6, against only 4% of the placebo. These findings supported the efficacy of this drug in reducing proptosis and CAS in pts with active GO Smith et al. (2017)
		-A randomized, double-masked, placebo-controlled, phase 3, multicenter trial, involved 83 pts with moderate to severe GO; 41 received teprotumumab and 42 placebo. Teprotumumab led to better outcomes in proptosis, CAS, diplopia, and quality of life than placebo; serious AEs were uncommon Douglas et al. (2020)
		The non responders were included in an extension of the phase 3 trial; they received teprotumumab as an open label, regardless of whether or not they had received the active drug, or placebo, during the 24-weeks treatment phase Smith. (2021). The response to the therapy occurs in a similar fraction of pts, such as in the initial intervention. Follow-up data (of phase 3 trial, plus extension study) revealed that the majority of pts who responded with amelioration of proptosis and diplopia at week 24 maintained their responses (56 and 58%, respectively) Smith. (2021)

AEs, Adverse Events; CAS, Clinical Activity Score; CS, Corticosteroids; GO, Graves’ Ophtalmopathy; ivMP, Intravenous Methylprednisolone; Pts, Patients; RA, Rheumatoid Arthritis; RTX, rituximab; TCZ, tocilizumab; TNF, Tumor Necrosis Factor TSAb, Thyroid Stimulating Antibodies; TSH-R, Thyroid-stimulating-hormone Receptor.

After the initial attempt of biologic therapies in GO (Antonelli et al., 1992; Baschieri et al., 1997), more recently, etanercept (that blocks the TNF-mediated inflammatory responses), TCZ (that acts against the IL-6 receptor), and RTX (that acts against CD20) have proven to be useful and safe therapeutic options in refractory GO treatment. Furthermore, teprotumumab (a human monoclonal anti-IGF-1R blocking antibody), has been revealed effective in the treatment of patients with moderate-severe GO and it is now approved for GO therapy in United States.

More, extensive researches are needed to deepen out these drugs as well as to identify new targeted and effective therapies, that will permit a more precise identification of GD, or GO, patients able to respond to specific targeted therapies.
